# Synthesis of Functional Building Blocks for Type III-B Rotaxane Dendrimer

**DOI:** 10.3390/polym13223909

**Published:** 2021-11-12

**Authors:** Chak-Shing Kwan, Watson K.-W. Ho, Yanyan Chen, Zongwei Cai, Ken Cham-Fai Leung

**Affiliations:** 1 State Key Laboratory of Environmental and Biological Analysis, Department of Chemistry, The Hong Kong Baptist University, Kowloon Tong, Kowloon, Hong Kong, China; 14485761@life.hkbu.edu.hk (C.-S.K.); 19482108@life.hkbu.edu.hk (Y.C.); zwcai@hkbu.edu.hk (Z.C.); 2 Department of Chemistry, The Chinese University of Hong Kong, Shatin, NT, Hong Kong, China; watsonho0910@gmail.com

**Keywords:** crown ether, fibroblast, functional dendron, macromolecular machine, rotaxane dendrimer

## Abstract

Second-generation type III-B rotaxane dendrons, equipped with succinimide and acetylene functional groups, were synthesized successfully and characterized by NMR spectroscopy and mass spectrometry. A cell viability study of a dendron with a normal cell line of L929 fibroblast cells revealed no obvious cytotoxicity at a range of 5 to 100 μM. The nontoxic properties of the sophisticated rotaxane dendron building blocks provided a choice of bio-compatible macromolecular machines that could be potentially developed into polymeric materials.

## 1. Introduction

Rotaxanes are a unique class of molecules that consist of one or multiple macrocycles that encircle one or more dumbbell-shaped threads. Such an entanglement between the macrocycles and threads in space through a non-covalent interaction is known as a mechanical bond [1], where these components cannot be separated until the chemical bond breaks or distorts within the atom of the components. Rotaxanes have been widely applied in molecular switching [2,3], molecular pumps [4,5,6], molecular motors [7], molecular machines [8] and organocatalysis [9,10]. Dendrimers are another class of highly ordered, hyperbranched macromolecules, composed of a repeating dendron unit with an exact molecular weight [11]. Typically, the molecular weight of dendrimers is higher than that of most drug molecules but smaller than that of linear polymers, in which such a molecular weight range can provide ample space for drug encapsulations in dendrimers’ near-spherical, three-dimensional morphology [12,13,14]. The fusion of rotaxane and dendrimers gives another, new class of mechanically interlocked macromolecules known as “Rotaxane Dendrimers”. The concept of rotaxane dendrimers was first proposed by Lee and Kim [15] in 2003, and was further elaborated on by Stoddart [1], Leung [16,17] and Yang [18]. Basically, there are three types of rotaxane dendrimers (I, II and III), while each type can be further subdivided into small categories (A, B and C). Type I and type II are the most abundant reported examples because of their structural simplicity and friendly preparation [19,20,21,22,23]. In the case of type III, it is defined as dendritic polyrotaxane, which means that the rotaxane mechanically bonds in a branch-like manner, similar to a dendrimer. Type III-A rotaxane dendrimers are defined as the mechanical bonds branching via the thread [24,25]. On the other hand, type III-B rotaxane dendrimers are defined as the mechanical bonds branching through the macrocycles [26]. Our group has reported a series of pure organic, metal-free higher-generation type III-B [26,27,28] and type III-C [29] rotaxane dendrimers, and demonstrated that such sophisticated macromolecules can act as drug carriers by encapsulating an anti-cancer drug (chlorambucil) [27] as well as actively releasing the drug from the carrier upon the acid–base switching mechanism (macromolecular machines). We also showed that such rotaxane dendrimers were non-inflammatory as well as non-toxic in mice bodies, and could be characterized by the mass spectrometric imaging technique [30].

The current synthetic method of producing higher-generation type III-B rotaxane dendrimers is based on the versatile copper-catalyzed azide-alkyne cycloaddition (CuAAC) [26,27,28] by using the building blocks of a relatively unstable pseudorotaxane instead of using a stable, pre-formed rotaxane. Pseudorotaxanes require relatively strong supramolecular interactions to maintain their interlocking structure between the thread and the macrocycle, subject to dissociation in harsh conditions. In order to expand the scope of synthetic methods for producing type III rotaxane dendrimers, we herein revisited the preparation of type III-B rotaxane second-generation (G2) dendrons with a *N*-hydroxysuccinimide (NHS) or an acetylene functional group with a molecular weight of approximately 5000 g/mol. Such functional dendrons can be the dendrimer building blocks, anchoring to other core materials such as nanoparticles [31,32], functional surfaces [33] or biomolecules through biorthogonal click chemistry or nucleophilic reactions [34]. It is necessary to assess the normal cell viability of such building blocks of rotaxane dendrimers before employing them as potential drug carriers [27,29] for cancer treatments [35,36,37,38,39], mainly based on enhancing the drug’s stability, lesion site targeting and prolonged drug release.

## 2. Materials and Methods

General. All chemicals, reagents and solvents were purchased without further purification from Sigma-Aldrich, St. Louis, MO, USA, unless otherwise stated. Deuterated solvents were purchased from Cambridge Isotope Laboratories, Tewksbury, MA, USA.

G2 rotaxane dendron-OSu **4**-3H·3PF_6_. G1 rotaxane dendron-acetylene **1**-H·PF_6_ (0.50 g, 0.26 mmol), ammonium thread diazide **3**-H·PF_6_ (0.058 g, 0.13 mmol) and functional crown ether **2** (0.075 g, 0.13 mmol) were dissolved in CH_2_Cl_2_ (5 mL, Labscan, Thailand). The resulting solution was degassed by sonication for 3 min. Cu(MeCN)_4_PF_6_ (0.09 g, 0.26 mmol), AcOH (32 μL, 0.52 mmol) and DIPEA (48 μL, 0.26 mmol) were added to the solution. The reaction mixture was stirred at an ambient temperature for 10 days. After that, a solution of AcOH (1 mL), CHCl_3_ (30 mL, Labscan, Thailand) and saturated Na_2_CO_3_ (15 mL) was added to the reaction mixture. The two layers were vigorously shaken in a separatory funnel until the organic layer became pale yellow and the aqueous layer became blue. The aqueous layer was extracted with CHCl_3_ (2 × 30 mL). The combined organic extracts were dried (MgSO_4_) and reprotonated by NH_4_PF_6_. The resulting solution was evaporated to dryness. Flash column chromatography with EtOAc on the silica gel of the residue gave a white solid. A second flash column chromatography with hexane, gradient to EtOAc, then to EtOAc/acetone (1:1), then to acetone with NH_4_PF_6_ (0.3 gL^−1^) on the silica gel of the residue gave G2 rotaxane dendron-OSu **4**-3H·3PF_6_ (0.18 g, 35%) as a white solid. M.p. = 158–159 °C. ^1^H NMR (CDCl_3_): 1.30 (s, 72 H, aliphatic H), 2.82 (s, 4 H, aliphatic H), 3.43 (m, 24 H, CH_2_O), 3.69 (m, 24 H, CH_2_O), 3.93 (m, 24 H, CH_2_O), 4.55 (s, 12 H, CH_2_NH_2_^+^), 4.71 (s, 4 H, CH_2_N), 4.97 (s, 16 H, CH_2_O), 5.39 (br, 20 H, CH_2_O), 6.58 (br, 7 H, ArH), 6.77 (s, 9 H, ArH), 7.05 (br, 12 H, ArH), 7.26 (br, 27 H, CONH/ArH), 7.33 (br, 18 H, ArH), 7.41 (br, 18 H, ArH), 7.60 (br, 6 H, NH_2_^+^) and 7.70 (br, 6 H, ArH). ^13^C NMR (CDCl_3_): *δ* = 25.74, 31.39, 34.65, 52.11, 53.31, 58.23, 67.78, 68.06, 70.23, 70.62, 77.37, 107.28, 108.45, 111.33, 111.65, 112.39, 117.31, 121.11, 121.69, 124.75, 125.63, 127.65, 128.27, 128.27, 129.61, 131.68, 131.82, 133.35, 136.06, 136.20, 136.52, 143.04, 146.09, 146.93, 147.09, 147.72, 149.80, 151.27, 153.11, 159.94, 166.15, 166.72 and 166.91. HRMS (ESI): C_261_H_304_N_24_O_46_ [M–3PF_6_+4H_2_O]^3+^: calcd 1503.4057; found 1503.5191.

G2 rotaxane dendron-acetylene **5**-3H·3PF_6_. To a solution of G2-rotaxane dendron-OSu **4**-3H·3PF_6_ (0.4 g, 0.08 mmol) in CH_2_Cl_2_ (1 mL), propagyl amine (0.1 mL, 1.78 mmol) was added. The reaction mixture was stirred at an ambient temperature for 12 h. After that, the solution was diluted by 15 mL CH_2_Cl_2_. The organic solution was dried (MgSO_4_) and reprotonated by NH_4_PF_6_. The solid was filtered and the solvents were evaporated to dryness. The resulting yellow oil was washed by EtOH (5 mL) twice to yield a pale-yellow solid. The resulting while solid was washed by EtOH, and G2 rotaxane dendron-acetylene **5**-3H·3PF_6_ (0.20 g, 50%) was obtained as a pale-yellow solid. M.p. = 156–157 °C. ^1^H NMR (CDCl_3_): 1.30 (s, 72 H, aliphatic H), 2.19 (br, 1 H, aliphatic H), 3.40 (m, 24 H, CH_2_O), 3.60 (m, 24 H, CH_2_O), 3.93 (m, 24 H, CH_2_O), 4.04 (br, 2 H, aliphatic H), 4.55 (br, 12 H, CH_2_NH_2_^+^), 4.72 (br, 4 H, CH_2_N/CH_2_O), 4.96 (s, 16 H, CH_2_O), 5.39 (br, 20 H, CH_2_N/CH_2_O), 6.39 (d, J = 7.2 Hz, 1 H, ArH), 6.58 (m, 8 H, ArH), 6.77 (s, 10 H, ArH), 7.05 (br, 15 H, ArH), 7.20 (m, 20 H, CONH/ArH), 7.33 (d, J = 8.24 Hz, 19 H, ArH), 7.38 (d, J = 8.24 Hz, 19 H, ArH), 7.60 (br, 6 H, NH_2_^+^) and 7.77 (s, 6 H, ArH). ^13^C NMR (CDCl_3_): 29.50, 31.36, 34.612, 52.08, 53.29, 58.21, 67.75, 68.03, 70.19, 70.54, 77.37, 107.23, 108.46, 111.35, 111.67, 112.34, 121.64, 124.68, 125.59, 126.71, 127.62, 128.27, 129.61, 131.66, 131.79, 133.32, 136.06, 143.01, 146.06, 147.04, 147.70, 149.61, 149.81, 151.23, 159.90, 166.11 and 166.78 (3 signals were missing/overlapping) HRMS (ESI): C_260_H_303_N_24_O_43_ [M–3PF_6_]^3+^: calcd 1483.0754; found 1483.0764.

In vitro study. Mouse fibroblast L929 cells were obtained from the American Type Culture Collection (ATCC, Manassas, VA, USA) and cultured with DMEM (Invitrogen, Carlsbad, CA, USA) containing 10% fetal bovine serum (FBS), penicillin (100 U/mL) and streptomycin (100 μg/mL), at 37 °C in a humidified 5% CO_2_ atmosphere. Ten thousand L929 cells were seeded into the wells of a 96-well plate. After 12 h of incubation, the medium in the wells was replaced with 100 μL of fresh medium containing a different concentration of tested compounds. After 24 h of incubation, the medium was replaced with 100 μL of fresh medium containing 0.5 mg/mL MTT (Sigma-Aldrich, St. Louis, MO, USA). After 3 h of incubation the medium was removed, and formazan crystals were dissolved with DMSO (150 μL) for 10 min on a shaker. The absorbance of each well was measured by a Multiskan GO UV/Vis microplate spectrophotometer (Thermo Fisher Scientific, Waltham, MA, USA) at a wavelength of 540 nm.

## 3. Results and Discussion

**Figure 1:**
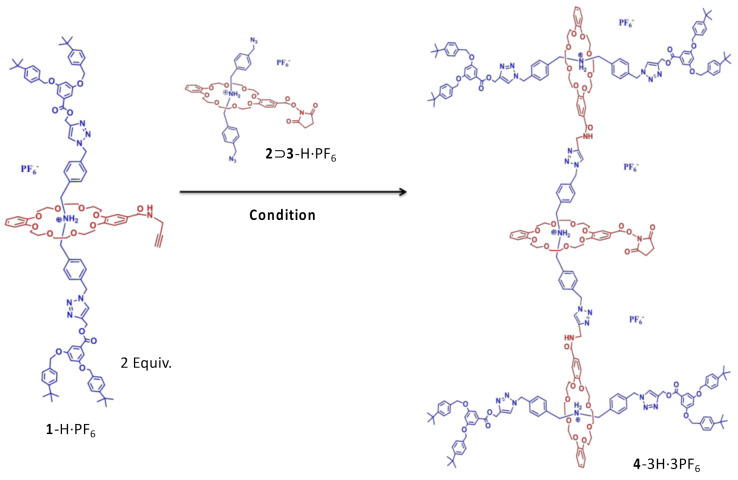
With G1 rotaxane dendron-acetylene **1**-H·PF_6_, double CuAAC reactions with pseudorotaxane [**2**⊃**3**-H·PF_6_] yielded G2 rotaxane dendron-OSu **4**-3H·3PF_6_. Condition: Cu(MeCN)_4_PF_6_ (2 equiv.), DIPEA (2 equiv.), AcOH (4 equiv.) and CH_2_Cl_2_. R.t., 10 days, 35%.

The basic components of these type III-B rotaxane dendrimers included NHS-functionalized crown ether and stopper Fréchet-type dendrons; azide-ended dibenzylammonium pseudorotaxane was prepared according to our previous report [26]. The G1 rotaxane dendron-acetylene **1**-H·PF_6_ was synthesized from a series of reactions through CuAAC. Two equivalents of the G1 rotaxane dendron-acetylene **1**-H·PF_6_ were then reacted with the dibenzylammonium-dibenzo [24] crown-8-based, mechanically interlocked pseudorotaxane [**2**⊃**3**-H·PF_6_] to give the G2 rotaxane dendron-OSu **4**-3H·3PF_6_ in a 35% yield through double CuAAC reactions with Cu(MeCN)_4_PF_6_ (Figure 1). The pseudorotaxane [**2**⊃**3**-H·PF_6_] was prepared by pre-mixing the dibenzo [24] crown-8-NHS compound **2** and the dibenzylammonium-diazide compound **3**-H·PF_6_, with an equal molar ratio in the solvent (20 mM). Noticeably, the pseudorotaxane was stable in slightly acidic conditions during the reaction; thereby, four equivalents of acetic acid (AcOH) plus two equivalents of diisopropylethylamine (DIPEA) were used.

**Figure 2:**
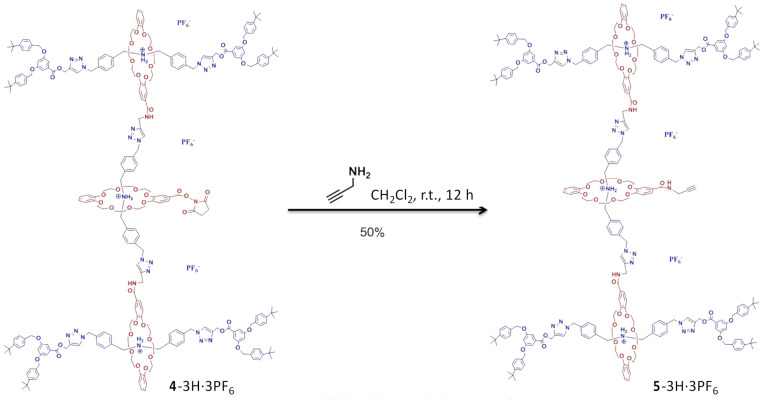
Synthesis of G2 rotaxane dendron-acetylene **5**-3H·3PF_6_.

**Figure 3:**
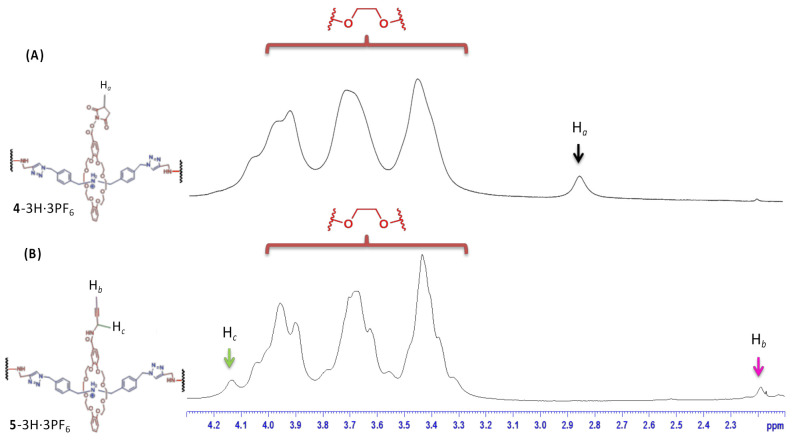
Partial ^1^H NMR spectra (2.1 to 4.3 ppm) of G2 rotaxane dendron-OSu **4**-3H·3PF_6_ and (**A**) G2 rotaxane dendron-acetylene **4**-3H·3PF_6_; (**B**) G2 rotaxane dendron-acetylene **5**-3H·3PF_6_.

Followed by the introduction of acetylene with propargylamine (Figure 2), the G2 rotaxane dendron-acetylene **5**-3H·3PF_6_ was successfully synthesized with a 50% yield from the G2 rotaxane dendron-OSu **4**-3H·3PF_6_. This reaction provided a clean and efficient functional group interconversion for the high-molecular-weight dendrons. The molecular structures of the two functional G2 rotaxane dendrons **4**-3H·3PF_6_ and **5**-3H·3PF_6_ were confirmed by ^1^H NMR spectroscopy. The observation (Figure 3A) of the proton signals (2.84 ppm) of the succinimide (H*_a_*) in the core of the G2 rotaxane dendron-OSu **4**-3H·3PF_6_ revealed the successful introduction of the NHS group of the G2 dendron (Figures S3 and S4), compared to the spectra (Figures S1 and S2) of **1**-H·PF_6_. On the other hand (Figure 3B), the disappearance of the succinimide proton (H*_a_*) and the appearance of the acetylene proton (H*_b_*) (2.19 ppm) and the propargyl proton (H*_c_*) (4.13 ppm) in the core of the G2 rotaxane dendron-acetylene **5**-3H·3PF_6_ (Figures S5 and S6) indicated the successful introduction of a propylene group to the G2 rotaxane dendron by a traditional acyl substitution reaction.

**Figure 4:**
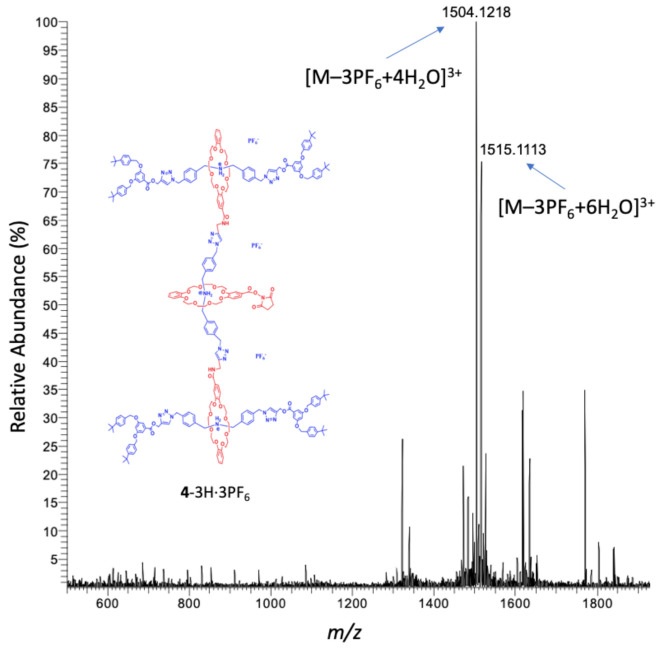
ESI-MS spectrum of the G2 rotaxane dendron-OSu **4**-3H·3PF_6_.

For characterization of the G2 rotaxane dendron-OSu **4**-3H·3PF_6_, the electrospray ionization mass spectrum (ESI-MS) revealed (Figure 4) the most abundant molecular ion signal (*m*/*z* 1504.1218) as [M–3PF_6_+4H_2_O]^3+^, losing its three counter-anion PF_6_^–^, as the detectable cation species. The expanded molecular ion signal (Figure S7) revealed that the peak spacings were approximately 0.3333, which demonstrated a +3 charged ion. Furthermore, the patterns of the peaks were basically the same between the experimental result and the theoretical analysis. Another molecular ion signal (*m*/*z* 1515.1113) can be attributed as [M–3PF_6_+6H_2_O]^3+^. Both ions revealed a dendron with a H_2_O adduct in the gas phase.

**Figure 5:**
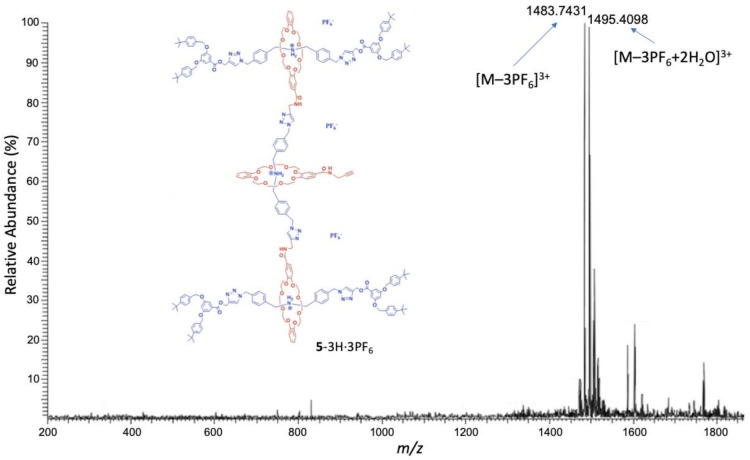
ESI-MS spectrum of the G2 rotaxane dendron-acetylene **5**-3H·3PF_6_.

For characterization of the G2 rotaxane dendron-acetylene **5**-3H·3PF_6_, the ESI-MS revealed (Figure 5) the most abundant molecular ion signal (*m*/*z* 1483.7431) as [M–3PF_6_]^3+^, losing its three counter-anion PF_6_^–^, as the detectable cation species. Another molecular ion signal (*m*/*z* 1495.4098) can be attributed as the molecular ion [M–3PF_6_+2H_2_O]^3+^. Overall, water adducts to two dendrons in the gas phase were observed through mass spectrometric analyses. 

**Figure 6:**
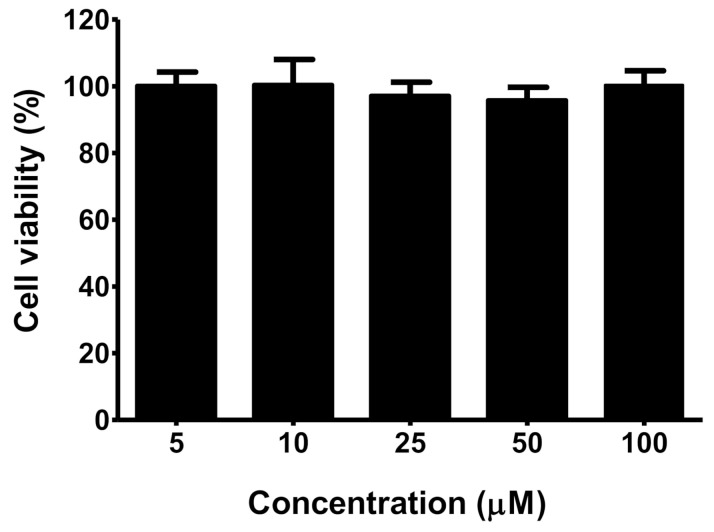
Cell viability of L929 cells as determined by an MTT assay after incubation with different concentrations of the dendron for 24 h. Data are expressed as means ± SD from four experiments.

On the other hand, an in vitro study of the new dendron was performed with L929 mouse fibroblast cells to shed light on cytotoxicity towards cells for extracellular matrix and collagen biosynthesis. To our delight, the tested dendron had no obvious cytotoxicity in L929 cells, even at the highest concentration of 100 μM (Figure 6).

## 4. Conclusions

In conclusion, novel type III-B second-generation rotaxane dendrons equipped with succinimide and acetylene functional groups were successfully synthesized and characterized. The new dendron compound did not reveal cytotoxicity with L929 fibroblast cells, even at a high concentration of 100 μM of the normal cells. The nontoxic nature as well as their sophisticated chemical structures would be essential criteria for developing next-generation macromolecular machines for potential biomedical use.

## Data Availability

The data presented in this study are available on request from the corresponding authors.

## Supplementary Materials

The following are available online at https://www.mdpi.com/article/10.3390/polym13223909/s1. Figure S1. ^1^H NMR spectrum of the G1 rotaxane dendron-acetylene **1**-H·PF_6_; Figure S2. 13C NMR spectrum of the G1 rotaxane dendron-acetylene **1**-H·PF_6_; Figure S3. ^1^H NMR spectrum of the G2 rotaxane dendron-OSu **4**-3H·3PF_6_; Figure S4. 13C NMR spectrum of the G2 rotaxane dendron-OSu **4**-3H·3PF_6_; Figure S5. ^1^H NMR spectrum of the G2 rotaxane dendron-acetylene **5**-3H·3PF_6_; Figure S6. 13C NMR spectrum of the G2 rotaxane dendron-acetylene **5**-3H·3PF_6_; Figure S7. Expanded mass spectrum of the molecular ion signal of the G2 rotaxane dendron-OSu **4**-3H·3PF_6_ and comparison between the experimental result (top) and the theoretical analysis (bottom).
