# Supplementary material for: Synthesis of Functional Building Blocks for Type III-B Rotaxane Dendrimer
Source: Polymers (Basel). 2021 Nov 12;13(22):3909. doi: 10.3390/polym13223909 (PMC8622516; doi:10.3390/polym13223909)
Supplement: Supplementary file 1 [file polymers-13-03909-s001.zip › polymers-1457711-supplementary.pdf]

Electronic Supplementary Information

Synthesis of Functional Building Blocks for Type III-B Rotaxane Dendrimer

Chak-Shing Kwan,[1] Watson K.-W. Ho,[2] Yanyan Chen,[1] Zongwei Cai,[1] Ken Cham-Fai Leung*[1]

[1] State Key Laboratory of Environmental and Biological Analysis, Department of Chemistry, The Hong Kong Baptist University, Kowloon Tong, Kowloon, P. R. China.

[2] Department of Chemistry, The Chinese University of Hong Kong, Shatin, NT, P. R. China.

E-mail: cfleung@hkbu.edu.hk

1-H·PF_6

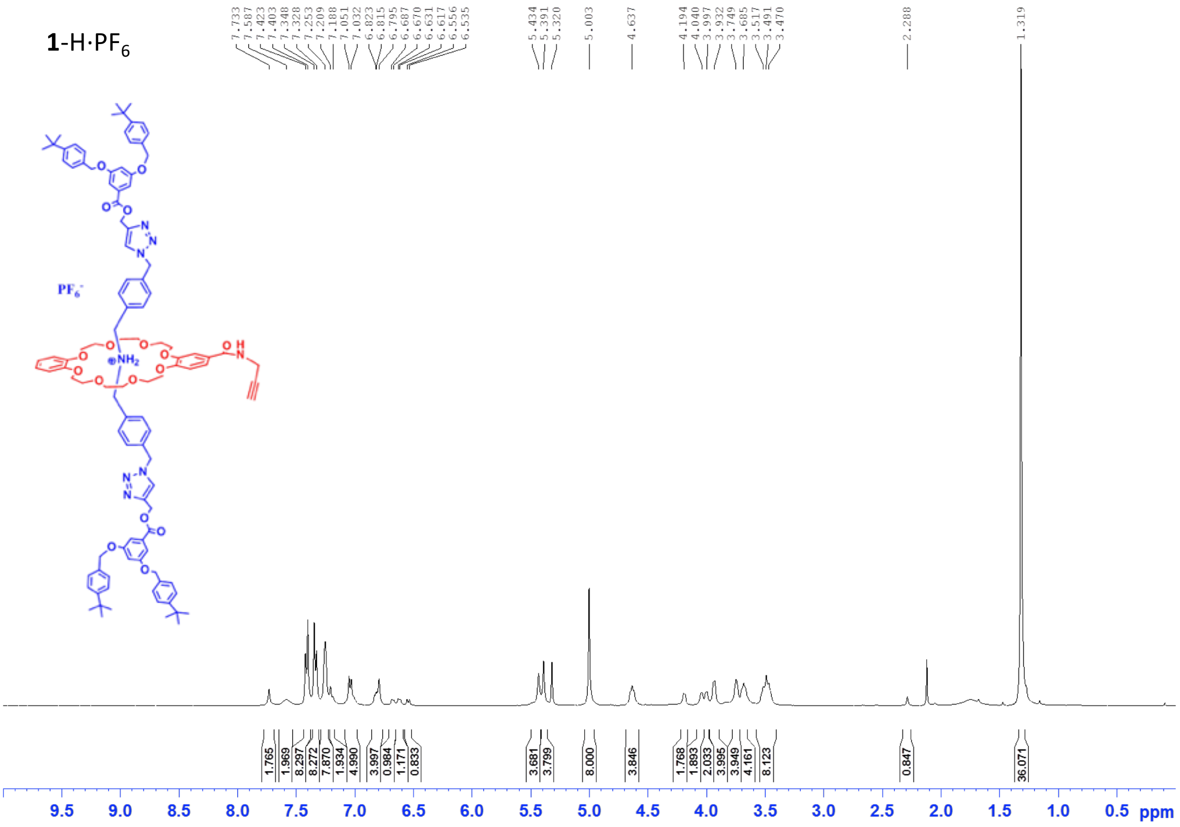

Figure S1. ^{1}H NMR spectrum of the G1 rotaxane dendron-acetylene **1**-H·PF_6.

1-H·PF_6

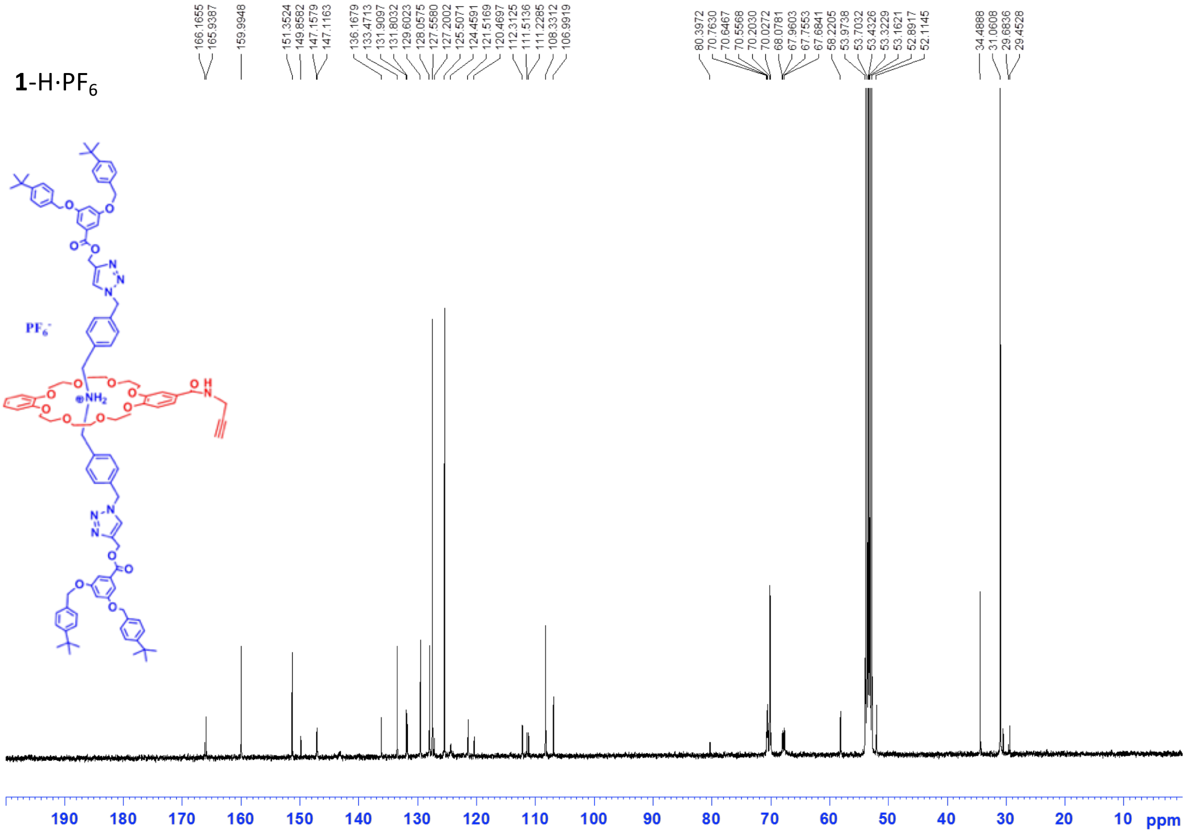

Figure S2. ^{13}C NMR spectrum of the G1 rotaxane dendron-acetylene **1**-H·PF_6.

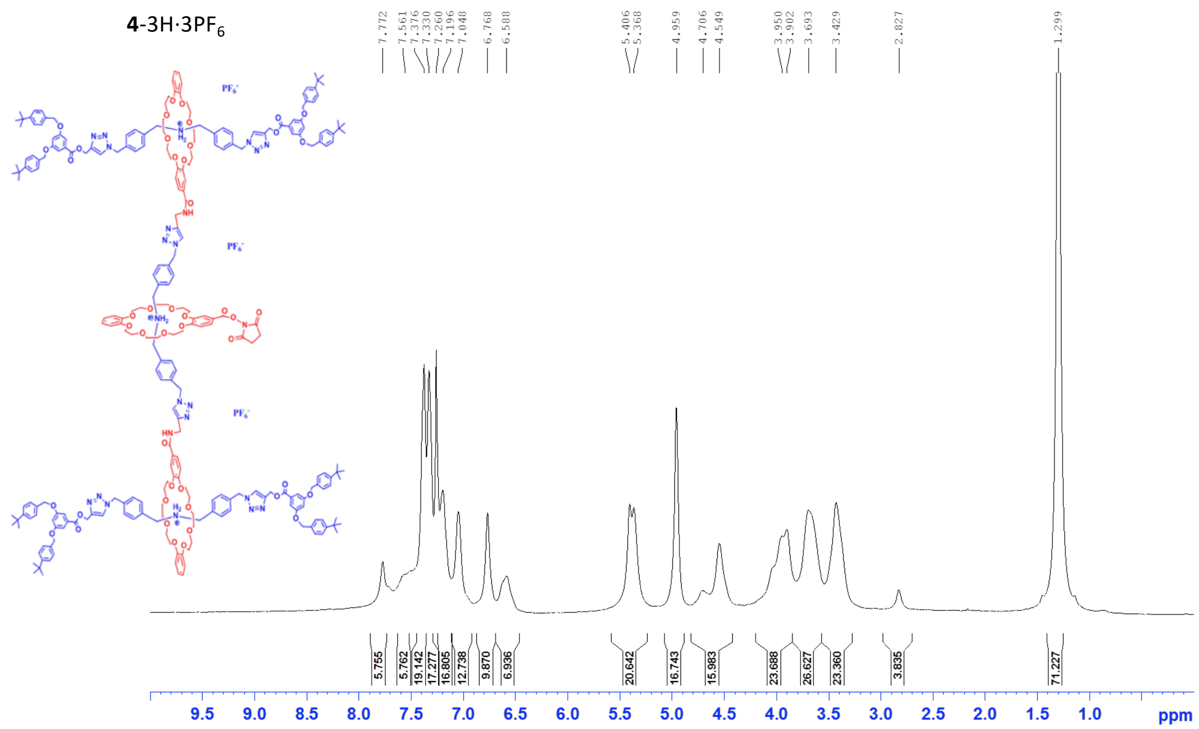

Figure S3. ^{1}H NMR spectrum of the G2 rotaxane dendron-OSu **4**-3H·3PF$_6$.

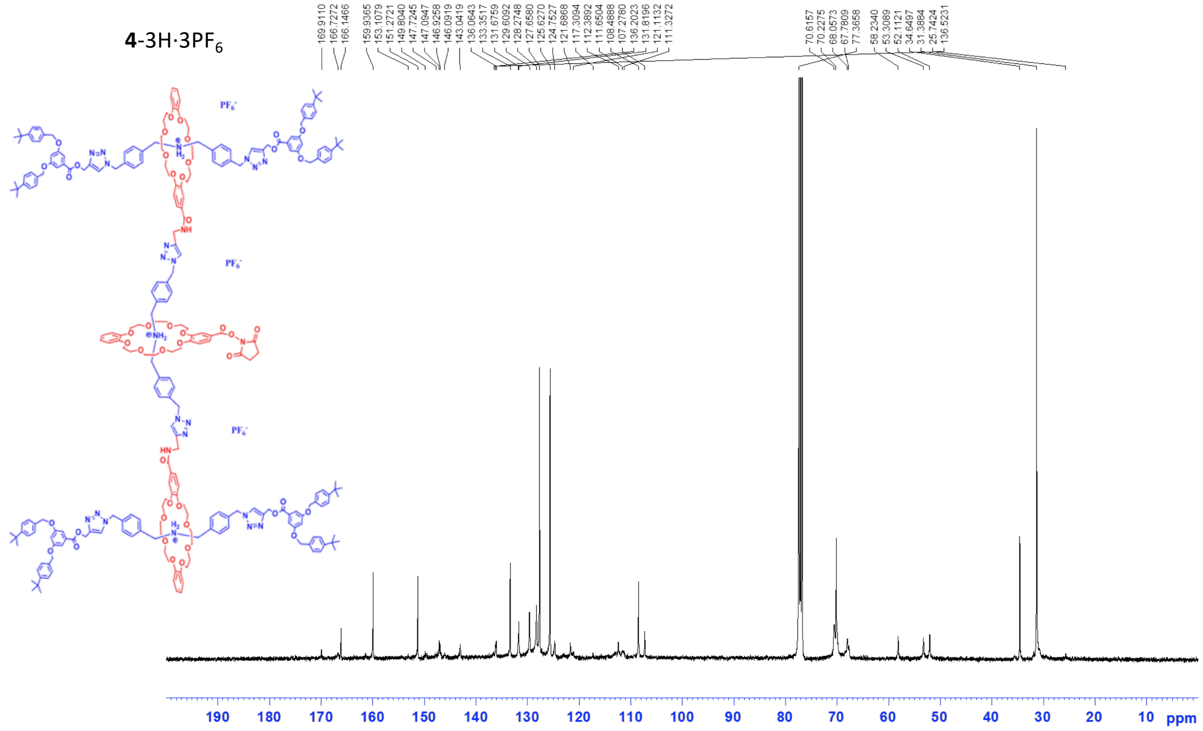

Figure S4. ^{13}C NMR spectrum of the G2 rotaxane dendron-OSu **4**-3H·3PF$_6$.

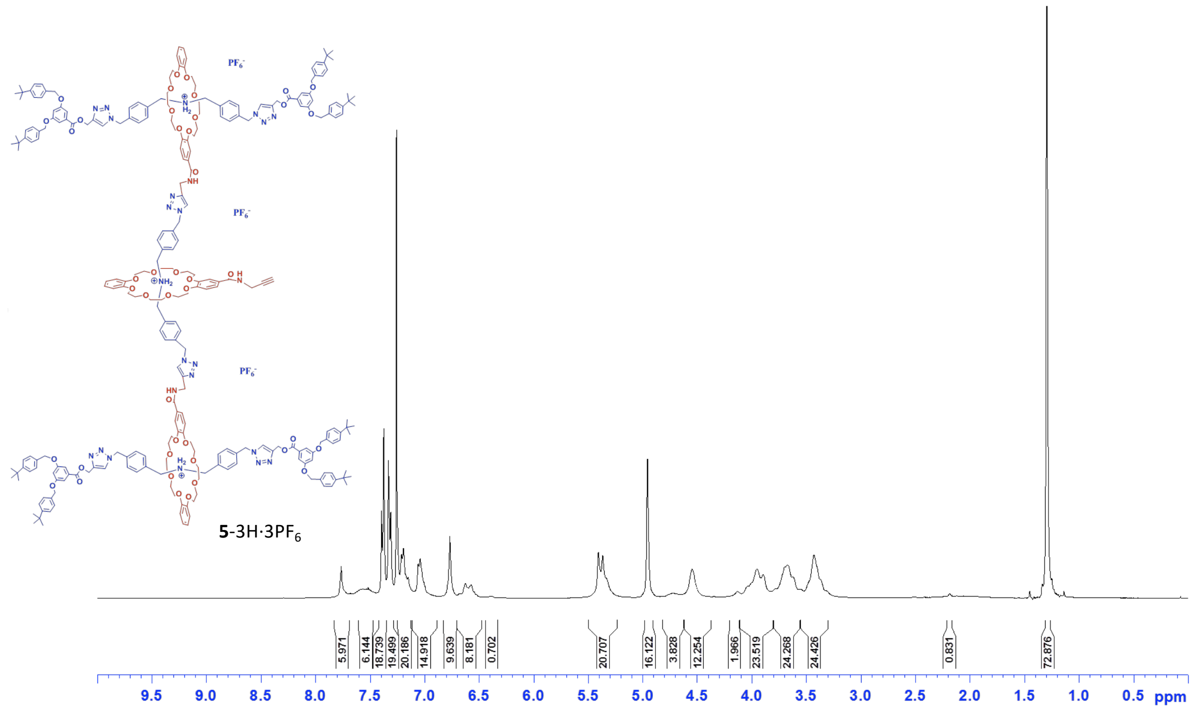

Figure S5. ^{1}H NMR spectrum of the G2 rotaxane dendron-acetylene **5**-3H·3PF$_6$.

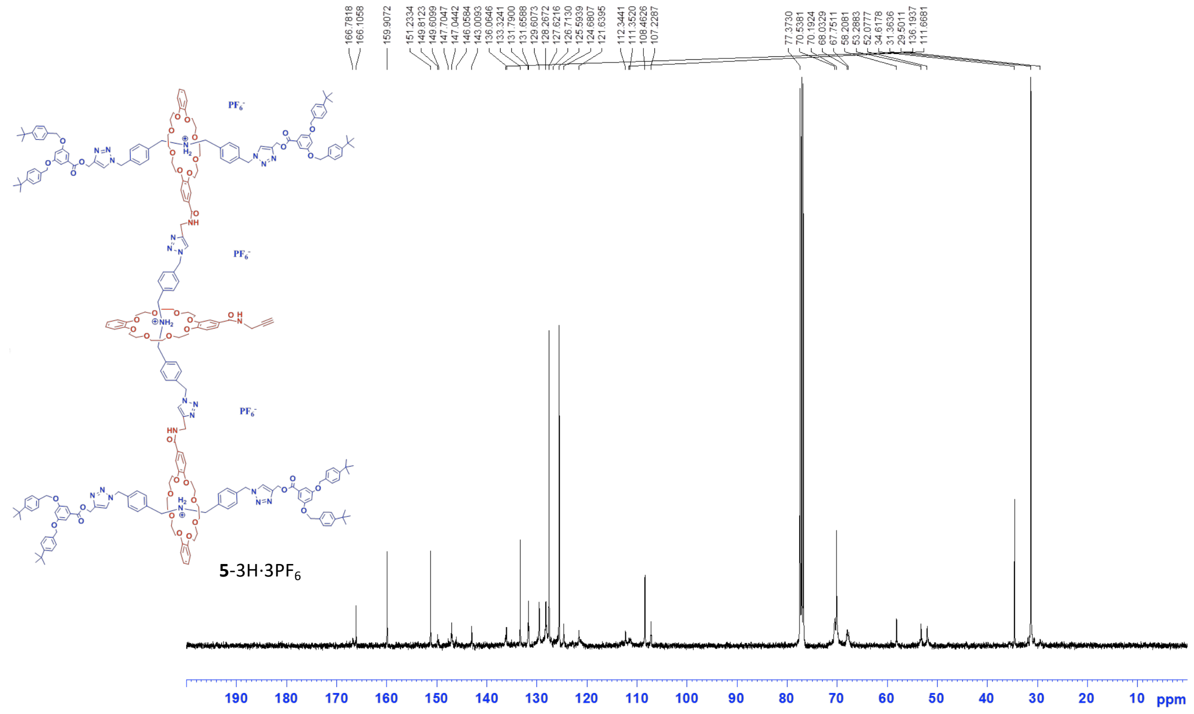

Figure S6. ^{13}C NMR spectrum of the G2 rotaxane dendron-acetylene **5**-3H·3PF$_6$.

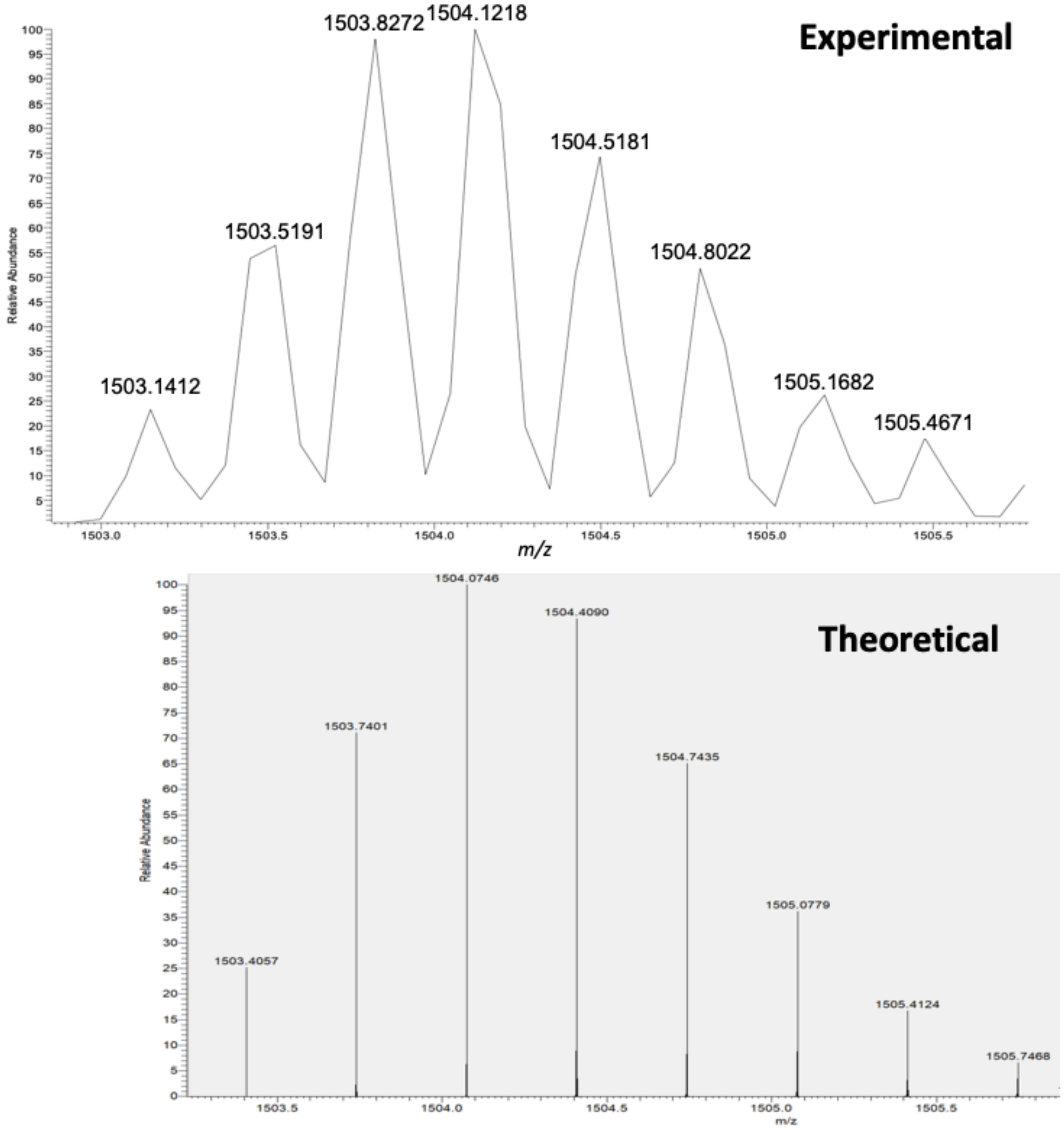

Figure S7. Expanded mass spectrum of the molecular ion signal of the G2 rotaxane dendron-OSu **4**-3H·3PF_6 and comparison between the experimental result (top) and the theoretical analysis (bottom).
